# Effect of Mannitol Dry Powder Challenge on Exhaled Nitric Oxide in Children

**DOI:** 10.1371/journal.pone.0054521

**Published:** 2013-01-18

**Authors:** Juerg Barben, Marie-Pierre F. Strippoli, Daniel Trachsel, Barbara Schiller, Juerg Hammer, Claudia E. Kuehni

**Affiliations:** 1 Department of Paediatric Pulmonology & Allergology, Children’s Hospital, St. Gallen, Switzerland; 2 Institute of Social and Preventive Medicine (ISPM), University of Bern, Bern, Switzerland; 3 Department of Pulmonology and Intensive Care, University Children’s Hospital Basel, Basel, Switzerland; University of Tübingen, Germany

## Abstract

**Background:**

Fractional exhaled nitric oxide (FENO), a non-invasive marker of eosinophilic airway inflammation, is increasingly used for diagnostic and therapeutic decisions in adult and paediatric asthma. Standardized guidelines for the measurement of FENO recommend performing FENO measurements before rather than after bronchial provocation tests.

**Objective:**

To investigate whether FENO levels decrease after a Mannitol dry powder (MDP) challenge in a clinical setting, and whether the extent of the decrease is influenced by number of MDP manoeuvres, baseline FENO, atopy and doctor diagnosed asthma.

**Methods:**

Children aged 6–16 years, referred for possible reactive airway disease to a respiratory outpatient clinic, performed an MDP challenge (Aridol®, Pharmaxis, Australia). FENO was measured in doublets immediately before and after the challenge test using the portable NIOX MINO® device (Aerocrine, Stockholm, Sweden). We analysed the data using Kruskal-Wallis rank tests, Wilcoxon signed rank tests and multivariable linear regressions.

**Results:**

One hundred and seven children completed both tests (mean±SD age 11.5±2.8 years). Overall, median (interquartile range) FENO decreased slightly by −2.5 ppb (−7.0, −0.5), from 18.5 ppb (10.5, 45.5) before the MDP challenge to 16.5 ppb thereafter (8.5, 40.5; p<0.001). In all participants, the change in FENO was smaller than one standard deviation of the baseline mean. The % fall in FENO was smaller in children with less MDP manoeuvres (e.g. higher bronchial responsiveness; p = 0.08) but was not influenced by levels of baseline FENO (p = 0.68), atopy (p = 0.84) or doctor diagnosed asthma (p = 0.93).

**Conclusion:**

MDP challenge test influences FENO values but differences are small and clinically barely relevant.

## Introduction

Asthma is the most common chronic disease of childhood, and its diagnosis remains clinical [1]. A diagnosis of asthma in children is usually based on history of a characteristic pattern of episodic respiratory symptoms and signs in the absence of an alternative explanation of them. In children, as in adults, tests of airflow obstruction, airway responsiveness and airway inflammation may provide support for a diagnosis of asthma [1]. The frequent finding of exercise-related airway narrowing in many children with asthma has been used as a diagnostic tool for years, especially when the diagnosis of asthma is uncertain [2]. Testing for exercise induced bronchoconstriction is not very sensitive, but highly specific for the diagnosis of pediatric asthma compared to direct bronchial provocation tests using methacholine [3].

Mannitol dry powder (MDP) is a new indirect bronchial provocation test, consisting of a hyperosmolar challenge comparable to hypertonic saline [4], [5]. In children, a MDP challenge is easier, quicker to perform and better tolerated than a challenge test with hypertonic saline or methacholine, and it shows comparable accuracy for diagnosing asthma as exercise testing [6]–[9]. For these reasons, it is likely to become one of the standard bronchial challenge tests in clinical practice and research [10].

Fractional exhaled nitric oxide (FENO), a non-invasive marker of eosinophilic airway inflammation, is increasingly used for diagnostic and therapeutic decisions in adult and paediatric asthma [11]. Standardized guidelines for the measurement of FENO, published by the European Respiratory Society (ERS) and the American Thoracic Society (ATS) [12], recommend to perform FENO measurements *before* rather than after exercise testing, because both spirometry and exercise affect FENO levels in children, particularly those with asthma [13], [14]. To plan the sequence of different tests for single children, it is important to know whether FENO levels are affected by a MDP challenge, similarly as by exercise tests.

The aim of this study was therefore to determine whether FENO levels decrease after a MDP challenge in a clinical setting. In addition, we assessed whether the extent of this decrease is affected by: baseline FENO, severity of bronchial hyperresponsiveness (BHR, which is inversely proportional to the number of MDP manoeuvres), atopy or a diagnosis of asthma.

## Methods

### Ethics Statement

All parents and children gave informed consent for the study. The local ethics committee and the Swiss agency for the authorisation and surveillance of therapeutic products (Swissmedic) approved the study.

### Subjects and Study Design

We studied 107 white children (Caucasian Europeans) aged 6 to 16 years, who had been referred for possible reactive airway disease to respiratory outpatient clinics in two tertiary children’s hospitals in Switzerland (St.Gallen, Basel). Details of the study have been published elsewhere [6]. Exclusion criteria were other respiratory diseases (e.g. cystic fibrosis, primary ciliary dyskinesia) and a respiratory tract infection during the preceding four weeks.

### Fractional Exhaled NO

We measured FENO in doublets immediately before and after the MDP challenge using the portable multi-gas analyzer (NIOX MINO®, Aerocrine, Sweden), in accordance with published guidelines [12] and previous studies using this device [15], [16]. The portable analyzer ensures a constant expiratory flow of 50±5 ml/s, has an accuracy of ±10% with a minimum of ±5 ppb. FENO measurements from this portable analyzer correlate well with those obtained by chemiluminiscence detectors [16].

### Mannitol Dry Powder Challenge

The MDP challenge (Aridol®, Pharmaxis, New South Wales, Australia) was performed as described previously [5], [7]. In brief, subjects were asked to inhale the contents of a MDP capsule through the delivery device (Osmohaler®). Doses were increased step-wise (0, 5, 10, 20, 40, 80, 160, 160, 160 mg) until there was either a ≥15% fall of forced expiratory volume in 1 second (FEV_1_) compared to baseline, or a 10% fall in FEV_1_ between two doses. After each dose, children were instructed to perform a 5-second breath-hold, and then spirometry was performed in duplicates one minute later, and the higher FEV_1_ was recorded. The results of the MDP challenge were reported as a percent decrease of FEV_1_ from baseline (FEV_1_ value measured after the 0-mg capsule), and the provocative dose inducing a 15% drop of FEV_1_ (PD_15_) was calculated. At the end of the challenge, a short**-**acting beta_2_-agonist was given to reverse bronchoconstriction. A negative MDP challenge, defined as no significant fall in FEV_1_ after a cumulative dose of 635 mg, included eleven FEV_1_ manoeuvres in doublets. BHR severity is defined as mild (155–635 mg, 8–10 manoeuvres), moderate (35–155 mg, 5–7 manoeuvres) and severe (<35 mg, ≤4 manoeuvres).

### Clinical Assessment

The responsible paediatric respiratory physicians made a diagnosis (asthma, or alternative diagnoses) for every child after the first visit, taking into consideration medical history, clinical examination and all conventional test results (including bronchodilator response) before MDP challenge and FENO measurement were performed.

### Skin Prick Test

We performed skin prick tests (SPT) using birch, grass, mugwort, alternaria, cat, house dust mites (*D. pteronyssinus*) and positive and negative controls, considering a wheal of >3 mm as positive. These allergens cover 95% of inhaled allergies in Switzerland [17]. Atopy is defined as mild (1 to 2 positive SPT) and moderate (≥3 positive SPT).

### Statistics

We analysed the data with STATA, version 11.2 (Stata Corporation, Austin, Texas). Because FENO levels were not normally distributed, we used non-parametric tests: the Kruskal-Wallis rank test to compare FENO levels between groups and the Wilcoxon signed rank test to compare FENO levels before and after MDP challenge. We assessed the association between % difference in FENO and levels of baseline FENO, doctor diagnosed asthma, atopy and BHR severity, using linear regressions.

## Results

One hundred and seven children completed both tests (mean±SD age 11.5±2.8 years). Eighty nine children (83%) were sensitized to at least one allergen and 71 (66%) to two or more, 64 (60%) were diagnosed by the clinician as having asthma, and 14 (13%) were on inhaled corticosteroids (**Table 1**). The MDP challenge was positive (BHR severity mild to severe) in 31 children (29%). Median (interquartile range IQR) FENO levels at baseline were higher in children diagnosed with asthma (26.0 (12.5,63.0) vs. 14.5 (8.0,22.5)), in atopic children (moderate 21.3 (12.5,56.5), mild 22.0 (12.5,49.0), none 8.0 (5.5,14.5)), and in those with a positive MDP challenge test (moderate to severe 65.0 (40.5,122.5), mild 42.5 (25.0,71.3), normal 14.0 (9.3,22.3); all p≤0.001).

**Table 1:** Patients characteristics (N = 107).

Characteristics	N (%)
Patients, No	107
**Anthropometric data**	
Male gender	66 (62%)
Age, yr *	12 (9–14; 6–17)
Height, cm *	147 (132–164; 115–182)
Weight, kg *	38 (29–54; 20–84)
**Clinical features**	
Doctor diagnosis of asthma	64 (60%)
More than 4 attacks of wheeze	38 (36%)
Use of inhaled corticosteroids	14 (13%)
**Atopy**	
None (negative SPT)	18 (17%)
Mild (1 to 2 positive SPT)	39 (36%)
Moderate (≥3 positive SPT)	50 (47%)
**Spirometry (at baseline)** *	
FVC, % predicted	99 (90–108; 65–128)
FEV_1_, % predicted	97 (88–104; 69–128)
FEV_1_/FVC	83 (76–88; 61–99)
MEF_50_, % predicted	75 (61–91; 29–126)
**BHR severity (MDP challenge)** †	
Normal (>635 mg, 11 manoeuvres)	76 (71%)
Mild (155–635 mg, 8–10 manoeuvres)	16 (15%)
Moderate to severe (<155 mg, ≤7 manoeuvres)	15 (14%)

Abbreviations: SPT, skin prick test; FVC, forced volume capacity; FEV_1_, forced expiratory volume in 1 sec; MEF_50_, maximal expiratory flow at 50%; BHR, bronchial hyperresponsiveness; MDP, mannitol dry powder.

* Results reported as median (interquartile range; range).

† BHR reported as MDP challenge results (provocative dose (in mg) inducing a 15% drop of FEV_1_ (PD_15_)). BHR severity is directly correlated with No of MDP manoeuvres.

**Figure 1:**
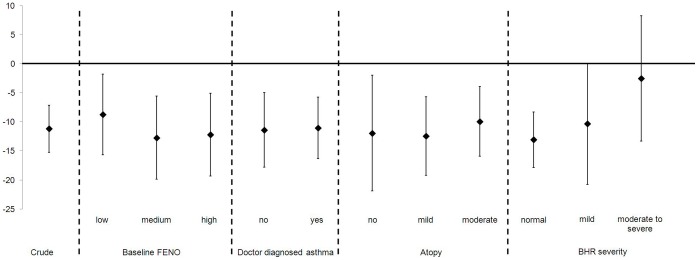
% difference of FENO after MDP challenge by baseline FENO, asthma, atopy and BHR severity. Abbreviations: FENO, fractional exhaled nitric oxide; MDP, mannitol dry powder; BHR, bronchial hyperresponsiveness; SPT, skin prick test. Baseline FENO defined as tertiles (low levels: <13 ppb; medium levels: 13 ppb–30 ppb; high levels: >30 ppb). Atopy defined as none (negative SPT), mild (1 to 2 positive SPT), moderate (≥3 positive SPT). BHR reported as MDP challenge results (provocative dose (in mg) inducing a 15% drop of FEV_1_ (PD_15_)) and defined as normal (>635 mg, 11 manoeuvres), mild (155–635 mg, 8–10 manoeuvres), moderate to severe (<155 mg, ≤7 manoeuvres).

Overall, median (IQR) FENO decreased by −2.5 ppb (−7.0, −0.5), from 18.5 ppb (10.5,45.5) before the MDP challenge to 16.5 ppb thereafter (8.5,40.5; p<0.001, **Table 2**). This change corresponds to a % difference of −11.2% (95% confidence interval −15.2, −7.1; **Figure 1**). In 81 children (76%) FENO decreased after the MDP challenge (median difference (IQR; range) −3.5 ppb (−8.5, −2.5; −27.5, −0.5)), in 18 (17%) children FENO levels increased (median difference 2.0 ppb (1.0,6.0; 0.5,9.5)) and in 8 (7%) they remained identical. In none of the children the change in FENO was larger than one standard deviation of the baseline mean. There was no association between % difference of FENO after MDP challenge and levels of baseline FENO (p = 0.68), doctor diagnosed asthma (p = 0.93) or presence of atopy (p = 0.84; **Figure 1**). However, the difference tended to be smaller in children who had moderate to severe BHR compared to those with normal BHR (p = 0.08; **Figure 1**).

**Table 2:** Levels of FENO at baseline and after mannitol dry powder challenge.

		FENO at baseline		FENO after MDP challenge			
	N	Median	IQR		Median	IQR	p-value†
**All children**	107	18.5	10.5–45.5		16.5	8.5–40.5	<0.001
**Doctor diagnosis of asthma**							
No	43	14.5	8.0–22.5		12.0	6.5–20.0	<0.001
Yes	64	26.0	12.5–63.0		23.3	10.3–56.8	<0.001
**Atopy**							
No (negative SPT)	18	8.0	5.5–14.5		6.8	5.0–12.0	0.006
Mild (1 to 2 positive SPT)	39	22.0	12.5–49.0		19.0	10.5–43.5	<0.001
Moderate (≥3 positive SPT)	50	21.3	12.5–56.5		21.3	9.5–46.5	<0.001
**BHR severity (MDP challenge)** *							
Normal (>635 mg, 11 manoeuvres)	76	14.0	9.3–22.3		12.3	7.3–20.8	<0.001
Mild (155–635 mg, 8–10 manoeuvres)	16	42.5	25.0–71.3		35.5	17.0–67.8	0.005
Moderate to severe (<155 mg, ≤7 manoeuvres)	15	65.0	40.5–122.5		64.5	46.5–102.5	0.079

Abbreviations: SPT, skin prick test; BHR, bronchial hyperresponsiveness; MDP, mannitol dry powder; FENO, fractional exhaled nitric oxide; IQR, interquartile range.

* BHR reported as MDP challenge results (provocative dose (in mg) inducing a 15% drop of FEV_1_ (PD_15_)). BHR severity is directly correlated with No of MDP manoeuvres.

† Non-parametric test, matched group = Wilcoxon signed rank test comparing medians of FENO at baseline and after MDP challenge.

## Discussion

This study showed that FENO levels are indeed slightly reduced after a MDP challenge, compared to baseline. The differences, although statistically significant, were however small and unlikely to affect clinical decisions relating to diagnosis or treatment.

Results were unaffected by baseline FENO levels, the presence of atopy or doctor diagnosed asthma. However, the decrease was larger in children with normal MDP challenge who had performed more MDP manoeuvres (11) compared to those with moderate to severe BHR with less MDP manoeuvres (≤7). This suggests that the increased ventilation associated with repeated MDP manoeuvres and associated spirometries might have caused the decrease. The median decrease (−2.5 ppb, IQR −7.0, −0.5) was comparable or a bit larger than the decrease reported for spirometry alone [3], [4], but smaller than the decrease reported for exercise tests (−5 ppb, from −23.5 ppb to −18.5 ppb) [13].

To our knowledge, this is the first study comparing FENO levels before and after MDP challenges. It includes a large number of children (107) and the clinical setting guarantees that results are representative for patients referred to respiratory outpatient clinics for potential reactive airways disease. Because most children were atopic and the majority diagnosed with asthma, results should not be extrapolated to healthy non-atopic children.

In conclusion, this study provides further support for the recommendation that FENO measurements in children, as a rule, should preferably be performed before spirometry and before bronchial provocation testing. This is particularly true for research. However, as the differences are small and clinically barely relevant, FENO measurements can still be interpreted even if performed after a MDP challenge test.
